# Supplementary material for: Rapid sensing of hidden objects and defects using a single-pixel diffractive terahertz sensor
Source: Nat Commun. 2023 Oct 25;14:6791. doi: 10.1038/s41467-023-42554-2 (PMC10600253; doi:10.1038/s41467-023-42554-2)
Supplement: Supplementary file 1 — Supplementary Information [file 41467_2023_42554_MOESM1_ESM.pdf]

Supplementary Information for

Rapid Sensing of Hidden Objects and Defects using a Single-Pixel Diffractive Terahertz Sensor

Jingxi Li[1,2,3], Xurong Li[1,3], Nezih T. Yardimci[1,3], Jingtian Hu[1,2,3], Yuhang Li[1,2,3], Junjie Chen[4], Yi-Chun Hung[1], Mona Jarrahi[1,3], and Aydogan Ozcan[1,2,3*]

[1]Electrical and Computer Engineering Department, University of California, Los Angeles, CA, 90095, USA

[2]Bioengineering Department, University of California, Los Angeles, CA, 90095, USA

[3]California NanoSystems Institute (CNSI), University of California, Los Angeles, CA, 90095, USA

[4]Physics & Astronomy Department, University of California, Los Angeles, CA, 90095, USA

[*]Correspondence to: ozcan@ucla.edu

Supplementary Figures

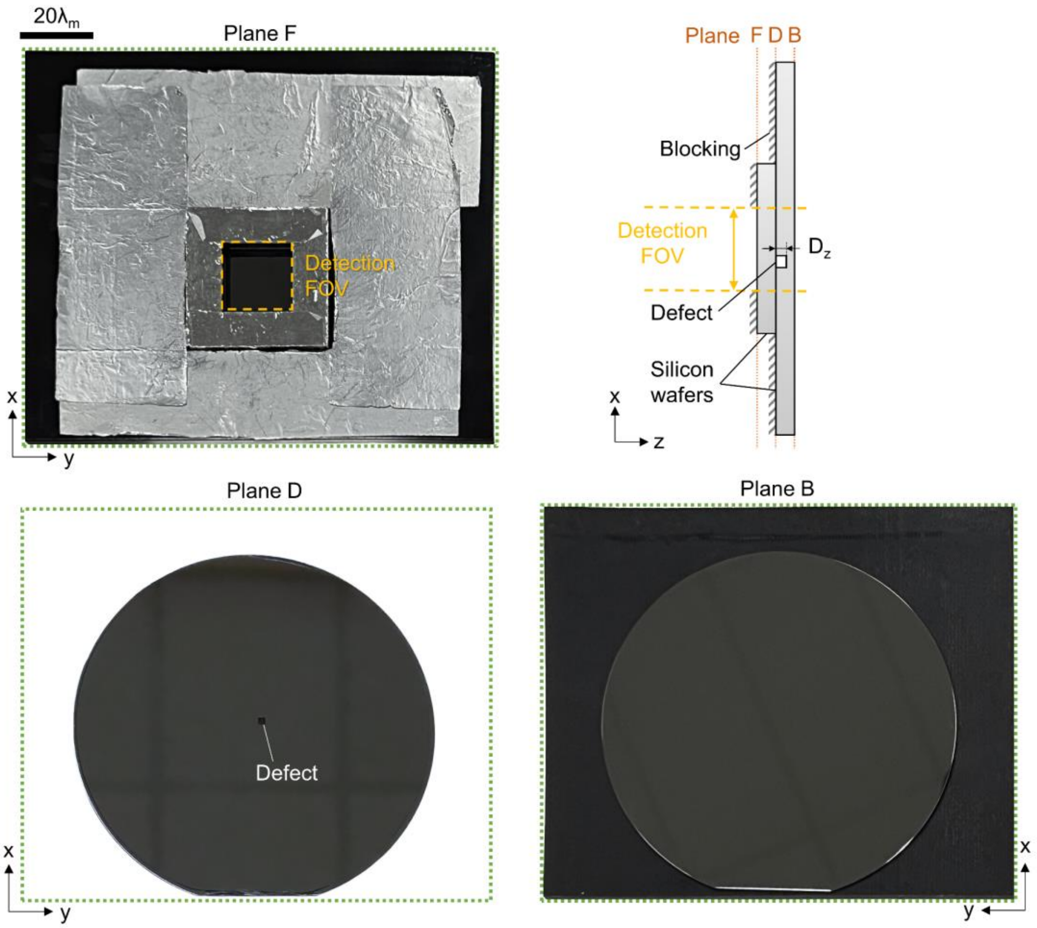

Figure S1. Photographs of the cross-sectional planes F, D and B of the test sample structure.

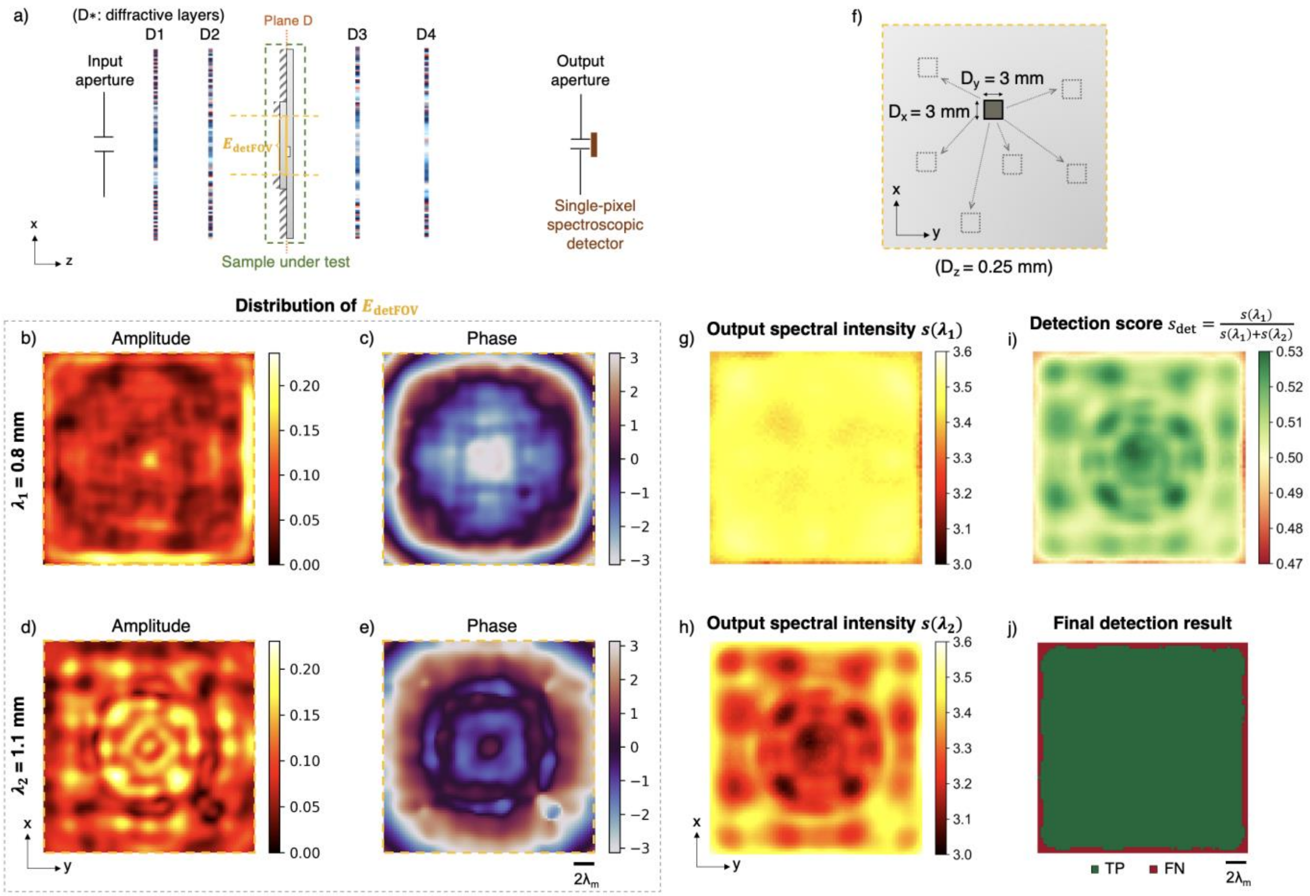

Figure S2. Analysis of the THz beam profile within the detection FOV and its impact on the defect detection results. a, Schematic of the diffractive detect sensor, showing the location of the terahertz field (E_{detFOV}) within the detection FOV. **b-e,** Amplitude and phase distributions of the field E_{detFOV} at two predetermined wavelengths, λ_1 and λ_2. **f,** Illustration used for analyzing the impact of the position of the hidden defect within the detection FOV. **g and h,** Output spectral intensity at the two operational wavelengths $s(\lambda_1)$ and $s(\lambda_2)$ as a function of the hidden defect's position within the detection FOV. **i,** Final detection score s_{det} as a function of the hidden defect's position within the detection FOV. **j,** Final detection results as a function of the hidden defect's position within the detection FOV. TP: True Positive. FN: False Negative.

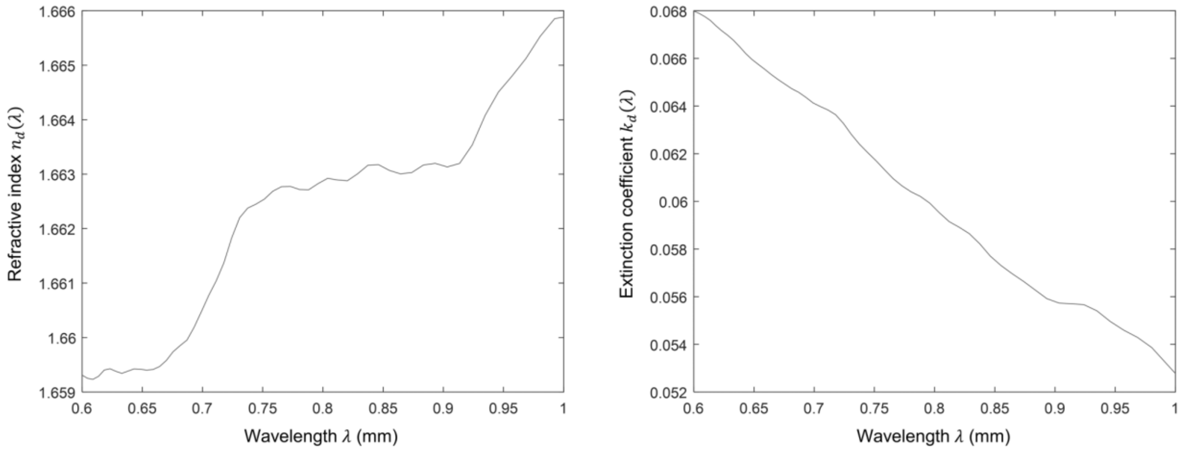

Figure S3. Dispersion curves of the material used for the diffractive layers: the refractive index $n_d(\lambda)$ (left) and the extinction coefficient $k_d(\lambda)$ (right).
